# Emergency units and COVID-19: Burnout, and empathy reported by nursing professionals and perceived by patients

**DOI:** 10.1590/0034-7167-2021-0869

**Published:** 2023-12-04

**Authors:** Daiane Silva Lopes Viana, Julia Yaeko Kawagoe

**Affiliations:** I Faculdade Israelita de Ciências da Saúde Albert Einstein. São Paulo, São Paulo, Brazil

**Keywords:** Empathy, Burnout, Professional, Emergency Medical Services, Nursing, COVID-19, Empatía, Agotamiento Profesional, Servicios Médicos de Urgencia, Enfermería, COVID-19, Empatia, Esgotamento Profissional, Serviços Médicos de Emergência, Enfermagem, COVID-19

## Abstract

**Objective:**

To investigate Burnout Syndrome and empathy self-reported by the nursing staff and empathy perceived by the patient.

**Method:**

Cross-sectional study in a public emergency unit in São Paulo (from October/2020 to March/2021). The nursing staff answered the Maslach Burnout Inventory and the Consultation and Relational Empathy Measure-Nurses (Brazilian version), whereas adult patients answered the Consultation and Relational Empathy Measure (Brazilian version). Descriptive and inferential analysis, with a 5% significance level.

**Results:**

A total of 92 professionals and 271 patients participated. Most professionals reported impact of COVID-19 (80; 86.96%) and, among them, increased Burnout Syndrome (93; 75%), but with low emotional exhaustion (71; 74%), low depersonalization (59; 78%) and high level of professional accomplishment (72; 83%). Most reported impact and increased empathy, and the results reported by professionals and patients (mean and standard deviation) were: 39.89 (6.44) and 38.25 (9.45), respectively.

**Conclusion:**

The professionals reported a low level of Burnout Syndrome and a high level of empathy in pandemic.

## RESUMO

**Objetivo:**

Investigar Síndrome de *Burnout* e empatia autorreferida pela equipe de enfermagem e empatia percebida pelo paciente.

**Método:**

Estudo transversal em Pronto-Socorro público de São Paulo (de outubro/2020 a março/2021). A equipe de enfermagem respondeu o *Maslach Burnout Inventory* e o *Consultation and Relational Empathy Measure-Nurses* (versão brasileira), e os pacientes adultos, o *Consultation and Relational Empathy Measure* (versão brasileira). Análise descritiva e inferencial, com nível de significância de 5%.

**Resultados:**

Participaram 92 profissionais e 271 pacientes. A maioria dos profissionais relatou impacto da COVID-19 (80; 86,96%) e, entre eles, aumento da Síndrome de *Burnout* (93; 75%), porém com baixa exaustão emocional (71; 74%), baixa despersonalização (59; 78%) e alto nível de realização profissional (72; 83%). A maioria relatou impacto e aumento da empatia, cujos resultados reportados pelos profissionais e pacientes (média e desvio-padrão) foram: 39,89 (6,44) e 38,25 (9,45), respectivamente.

**Conclusão:**

Os profissionais reportaram baixo nível da Síndrome de *Burnout* e alto nível de empatia na pandemia.

## RESUMEN

**Objetivo:**

Investigar el Síndrome de *Burnout* y la empatía auto-reportada por el personal de enfermería y la empatía percibida por el paciente.

**Método:**

Estudio transversal en un Servicio de Urgencias público de São Paulo (de octubre/2020 a marzo/2021). El personal de enfermería respondió al *Maslach Burnout Inventory* y al *Consultation and Relational Empathy Measure-Nurses* (versión brasileña), y los pacientes adultos, al *Consultation and Relational Empathy Measure* (versión brasileña). Análisis descriptivo e inferencial, con nivel de significancia del 5%.

**Resultados:**

Noventa y dos profesionales y 271 pacientes participaron. La mayoría de los profesionales informó impacto de la COVID-19 (80; 86,96%) y, entre ellos, aumento del Síndrome de *Burnout* (93; 75%), pero con bajo agotamiento emocional (71; 74%), baja despersonalización (59; 78%) y alto nivel de realización profesional (72; 83%). La mayoría refirió impacto y aumento de la empatía, cuyos resultados referidos por profesionales y pacientes (media y desviación estándar) fueron: 39,89 (6,44) y 38,25 (9,45), respectivamente.

**Conclusión:**

Los profesionales informaron bajo nivel de Síndrome de *Burnout* y alto nivel de empatía en la pandemia.

## INTRODUCTION

Burnout Syndrome has long been considered as a lasting stress related to work situations, which can be expressed by the word burnout^(1)^, and is included in the 11th Revision of the International Classification of Diseases (ICD-11) as an occupational phenomenon^(2)^.

Burnout Syndrome presents nonspecific manifestations by means of physical, psychological and behavioral symptoms^(3)^ and is characterized as a three-dimensional psychological syndrome: Emotional Exhaustion, Depersonalization in customer service and feelings of low personal accomplishment, being denominated, in this research, as Professional Accomplishment, by feelings of self-efficacy and accomplishment at work. The Maslach Burnout Inventory- Human Services Survey (MBI-HSS) instrument categorizes the intensity of Burnout Syndrome into low, medium or high level for each dimension, with high scores for Emotional Exhaustion and Depersonalization and low Professional Accomplishment determining the syndrome ^(4)^.

The COVID-19 (Coronavirus Disease 2019) pandemic, declared by the World Health Organization (WHO) in March 2020^(5)^, has impacted healthcare systems worldwide, especially the mental health of frontline healthcare workers^(6)^. Among these professionals, the nursing team is the largest contingent providing direct assistance to patients and stands out with high psychosocial risk^(7)^ in urgency and emergency services, herein called emergency units^(7-9)^. Emergency unist are 24/7 healthcare units where patients seek primary emergency care when no other service is available^(9)^.

Studies conducted before the COVID-19 pandemic already indicated risk factors for Emergency Unit nursing professionals to develop Burnout Syndrome, namely: inadequate working conditions, excessive number of patients, lack of material resources and inadequate physical structure, communication failure among multidisciplinary teams, among others^(10-12)^. Two systematic reviews with meta-analyses evidenced that emergency unit nurses presented high levels of Burnout Syndrome: high Emotional Exhaustion, Depersonalization and low Professional Accomplishment^(13-14)^.

With the COVID-19 pandemic, emergency units that had reduced observation bed capacity underwent significant changes in structure and workflow, resulting in overcrowded units with inpatients and critically ill patients^(15)^.

Under this perspective, nursing professionals working in emergency units started to live with increased risk of occupational infection and of developing Burnout Syndrome, due to factors arising from the pandemic: a new coronavirus and insecurity of the team as to proper clinical management; lack of adequate structure, adequate training and personal protective equipment (PPE); conflicting bonds with peers; execution of tasks that are often dramatic and distressing, in addition to dealing with the loss of patients, family members and professional colleagues^(8)^. On the other hand, high levels of Burnout Syndrome can negatively impact patient care, being associated with more errors, reduced patient satisfaction, decreased patient compliance to treatment recommendations and greater intention of the professional to leave the job^(14)^, generating possible conflicts, lack of humanization and reduced empathy^(16)^.

Empathy is defined as the ability to understand another person, their feelings and thoughts, and to transmit this understanding back to the person, in three ways: cognitive: intellectual ability to understand feelings; affective or emotional: ability to put oneself in another person’s place in a rational way; and behavioral: positive attitudes in understanding the critical situation experienced by the other person^(17-18)^.

Acting through effective communication, with the ability to perceive the other and his/her point of view^(19)^, that is, acting with professional empathy can be a coping strategy to prevent chronic professional stress and promote well-being^(20)^.

Furthermore, empathic and communication skills of professionals are essential to avoid unnecessary stress of patients and families in situations of pressure and uncertainty in emergency units, and thus improve patient satisfaction^(21)^. Therefore, Burnout Syndrome and empathy are closely linked and are relevant constructs for patientand professional-centered care.

Therefore, and because we have not identified studies with these themes in emergency units in the current pandemic, the guiding question of this study was: What is the impact of the COVID-19 pandemic on nursing professionals regarding the levels of Burnout Syndrome and empathic care provided in a public emergency unit?

## OBJECTIVES

To evaluate the impact of the COVID-19 pandemic on nursing professionals regarding Burnout Syndrome levels and empathic care provided in a public emergency unit.

## METHOD

### Ethical aspects

The project was approved by Ethics and Research Committees recognized by the National Research Ethics Commission, in compliance with the ethical norms and precepts established in Resolution 466/2012.

### Study design, period and setting

Cross-sectional study, with a quantitative approach, carried out from October 2020 to March 2021, in a medium-sized public emergency unit in the city of São Paulo. This research was guided by the tool Strengthening the Reporting of Observational Studies in Epidemiology (STROBE).

### Population and sample: Inclusion and exclusion criteria

The population was made up of 104 nursing professionals working in the adult observation room (nursing ward) and the patients they cared for. Inclusion criteria were: nurses, nursing technicians and nursing aides working in direct care for at least six months; exclusion criteria were: professionals on vacation and away on medical leave.

The sample of patients was of three patients for each nursing professional participating in the research. Inclusion criteria were: being over 18 years old, with preserved cognition and able to understand the text to answer the questions; and the exclusion criteria were: patients under observation for six hours or less and/or severe and/or unstable clinical condition.

### Study protocol

The eligible professionals were approached during working hours, when they were not in service and, after signing a free and informed consent form, they received a link, via WhatsApp, with a questionnaire for sociodemographic characterization and the instruments Maslach Burnout Inventory - Human Services Survey (MBI-HSS)^(22)^ and Consultation and Relational Empathy Measure (CARE Measure - Nurses), (Brazilian version)^(18)^, being at the participant’s discretion, the choice of the best place to answer them.

The MBI-HSS is a structured, self-applicable instrument developed by Christina Maslach and Susan E. Jackson^(22)^, with copyrights acquired by Mind Garden Inc. in 2010. A license to use the instrument was acquired for this research, and it was made available in Brazilian Portuguese by the publisher.

The MBI-HSS is made up of 22 items, distributed into three independent subscales. The Emotional Exhaustion subscale has nine items (1, 2, 3, 6, 8, 13, 14, 16, and 20), the Depersonalization subscale has five items (5, 10, 11, 15, and 22), and the Professional Accomplishment subscale, eight items (4, 7, 9, 12, 17, 18, 19, and 21). The evaluation of all items adopts a Likert-type scale ranging from 0 to 6, being: (0) never, (1) a few times a year or less, (2) once a month or less, (3) a few times a month, (4) once a week, (5) a few times a week, (6) daily. Each dimension is divided into three levels, according to the sum of the items: high, moderate, and low. For Emotional Exhaustion: ≥27: high level, from 19 to 26: moderate, and ≤18: low level; Depersonalization: ≥10: high level, from 6 to 9: moderate, and ≤5: low level; and Professional Accomplishment: ≥40: high level, from 34 to 39: moderate, and ≤33: low level^(22-23)^. At the end of the MBI-HSS instrument, there were questions about the impact of the COVID-19 pandemic on Burnout Syndrome levels, with Yes and No alternatives. If the answer was YES, there were two alternatives: increased or decreased.

The second instrument was the Consultation and Relational Empathy Measure Nurses (CARE Measure - Nurses), (Brazilian version), adapted and validated by Roberta Savieto et al. in 2019^(18)^, for self-evaluation of these professionals regarding empathy towards the care provided. The instrument contains 10 items, with six possibilities of evaluation each, with one “not applicable” and the other options ranging between “poor” and “excellent”, resulting in a score between 10 and 50, where 10 means unsatisfactory empathic behavior and 50, highly satisfactory^(18)^. At the end of the CARE Measure - Nurses instrument, there were questions about the impact of the COVID-19 pandemic on empathic care, with Yes and No alternatives. If the answer was YES, there were two alternatives: increased or decreased.

Eligible patients were approached and invited to participate in the research individually. After the acceptance and signature of the informed consent form, the researcher holding a tablet, next to the patient’s bed, asked the question and the patient answered, avoiding contact between the patient and the tablet. Thus, the patient answered the sociodemographic questionnaire questions and the CARE Measure instrument (Brazilian version) about the care provided by the nurse, nursing technician, or nursing aide who had provided care.

The Consultation and Relational Empathy (CARE) instrument - Brazilian version -, translated and validated by et al.^(24)^, consists of 10 items, with 6 possibilities of evaluation each, with one “not applicable” and the other options varying between “poor” and “excellent”, resulting in a score between 10 and 50, where 10 means unsatisfactory empathic behavior and 50, highly satisfactory.

All data were collected using the REDCap platform^(25-26)^. Biosafety protocols were followed during data collection: disinfection of materials and equipment and hand hygiene before and after participation in the study.

### Analysis of results and statistics

A 10% loss was estimated to constitute the sample of nursing professionals, with a correlation coefficient of 0.3 and a power of 91.3%, with the aid of the pwr package^(27)^.

Numerical variables were described by mean and standard deviation or median and interquartile range [IIQ (minimum and maximum)], and qualitative variables were described by absolute and relative frequency^(28)^. The association between perceived empathy and Burnout Syndrome categories was assessed by a generalized linear model with gamma distribution^(29)^. Chi-square test was applied for qualitative variables and Analysis of Variance (Anova) for quantitative variables, to verify the association between sociodemographic variables of the professionals and to verify the association between questions regarding the COVID-19 pandemic and the dimensions of the MBI-HSS among the categories of professionals^(30)^.

The Kruskal-Wallis test was applied to compare the mean scores obtained by the CARE Measure and the CARE Measure-Nurses, by professional category^(30)^. Sperman’s correlation coefficient was used to correlate the scores of the MBI-HSS dimensions and the CARE Measure and CARE Measure-Nurses; to calculate the correlation, the mean of patients per professional was used for the indicator “empathic behavior reported by patients”^(30)^_._ For all analyses, a significance level of p = 0.05 was adopted. The analyses were performed with SPSS software^(30)^.

## RESULTS

Ninety-four nursing professionals (eligible) participated in the research, but two were excluded because no patient was interviewed. All the analyses considered: 92 professionals, namely 23 nurses (25%), 28 nursing technicians (30.43%), and 41 nursing assistants (44.57%), and 271 patients who were assisted by these professionals.

### Sociodemographic characteristics of nursing professionals and patients

Table 1 presents the sociodemographic characterization of nursing professionals by professional category. The overall mean age (standard deviation) was 42.07 (7.81) years. Most participants were female (79; 85.87%), white (45; 48.91%), and mixed race (31; 33.70%), working during the daytime (57; 61.96%) and with a time of graduation of “11 years or more” (60; 65.22%). About the time of work in the emergency unit - study setting - most professionals had worked, in general, from “6 months to 5 years” (46; 50%), especially nurses and nursing technicians: 15 (65.22%) and 21 (75.00%), respectively (p=<0.001). Most had CLT jobs (77; 83.70%) and had no other employment in health care (52; 56.52%).

**Table 1:** Sociodemographic characteristics of nursing professionals, by professional category, in the Emergency Unit, São Paulo, São Paulo, Brazil, 2021

Variable	Professional Category				*p* value
	Nurse	Nursing technician	Nursing aide	Total	
Mean age (standard deviation)	41.17 (7.91)	39.52 (7.20)	44.30 (7.70)	42.07 (7.81)	0.063
Sex n (%)					
Female	20 (86.96)	22 (78.57)	37 (90.24)	79 (85.87)	0.387
Male	3 (13.04)	6 (21.43)	4 (9.76)	13 (14.13)	
Race/skin color n (%)					
White	13 (56.52)	13 (46.43)	19 (46.34)	45 (48.91)	0.746
Black	2 (8.70)	5 (17.86)	9 (21.95)	16 (17.39)	
Brown	8 (34.78)	10 (35.71)	13 (31.71)	31 (33.70)	
Work shift n (%)					
Day	14 (60.87)	20 (71.43)	23 (56.10)	57 (61.96)	0.433
Night	9 (39.13)	8 (28.57)	18 (43.90)	35 (38.04)	
Length of profession (%)					
6 months to 5 years	4 (17.39)	6 (21.43)	0 (0.00)	10 (10.87)	0.125
6 to 10 years	5 (21.74)	7 (25.00)	10 (24.39)	22 (23.91)	
11 to 14 years	4 (17.39)	6 (21.43)	11 (26.83)	21 (22.83)	
15 years or more	10 (43.48)	9 (32.14)	20 (48.78)	39 (42.39)	
Length of activity in the emergency unit - study setting n (%)					
6 months to 5 years	15 (65.22)	21 (75.00)	10 (24.39)	46 (50.00)	<0.001
6 to 10 years	3 (13.04)	5 (17.86)	14 (34.15)	22 (23.91)	
11 to 14 years	0 (0.00)	1 (3.57)	8 (19.51)	9 (9.78)	
15 years or more	5 (21.74)	1 (3.57)	9 (21.95)	15 (16.30)	
Type of employment bond to the emergency unit n (%)					
CLT	18 (78.26)	27 (96.43)	32 (78.05)	77 (83.70)	0.092
Statutory	5 (21.74)	1 (3.57)	9 (21.95)	15 (16.30)	
Has another employment bond in the healthcare field? n (%)					
No	14 (60.87)	15 (53.57)	23 (56.10)	52 (56.52)	0.870
Yes	9 (39.13)	13 (46.43)	18 (43.90)	40 (43.48)	

*n: number; CLT: Consolidation of Labor Laws; SP: São Paulo*

Table 2 presents the sociodemographic characterization of patients seen by professional category. The median age was 54.10; IIQ: 38.00 - 66.60, and most of them were male (158; 58.30%), white (145; 53.51%), with an education level of “up to complete elementary school” (155; 57.20%). The service was performed mainly during daytime (170; 62.73%) and in the nursing ward (168; 61.99%).

**Table 2:** Sociodemographic characteristics of patients, by professional category of the provider, in the Emergency Unit, São Paulo, São Paulo, Brazil, 2021

Variable	Professional Category				*p* value
	Nurse	Nursing technician	Nursing aide	Total	
Mean age	49.60	52.50	54.75	54.10	0.490
(IQR)	(35.80; 66.60)	(36.60; 68.50)	(38.60; 66.20)	(38.00; 66.60)	
Sex n (%)					
Female	25 (37.88)	36 (43.37)	52 (42.62)	113 (41.70)	0.765
Male	41 (62.12)	47 (56.63)	70 (57.38)	158 (58.30)	
Race/skin color n (%)					
White	37 (56.06)	43 (51.81)	65 (53.28)	145 (53.51)	0.205
Black	5 (7.58)	17 (20.48)	15 (12.30)	37 (13.65)	
Brown	24 (36.36)	23 (27.71)	42 (34.43)	89 (32.84)	
Level of education n (%)					
No education	0 (0.00)	8 (9.64)	5 (4.10)	13 (4.80)	0.179
Incomplete Elementary	29 (43.94)	28 (33.73)	51 (41.80)	108 (39.85)	
Complete Elementary	8 (12.12)	11 (13.25)	15 (12.30)	34 (12.55)	
Incomplete High School	5 (7.58)	8 (9.64)	18 (14.75)	31 (11.44)	
Complete High School	19 (28.79)	24 (28.92)	30 (24.59)	73 (26.94)	
Complete Higher Education	5 (7.58)	4 (4.82)	3 (2.46)	12 (4.43)	
Time of service n (%)					
Day	37 (56.06)	60 (72.29)	73 (59.84)	170 (62.73%)	0.085
Night	29 (43.94%	23 (27.71)	49 (40.16)	101 (37.27%)	
Accommodation n (%)					
Nursing ward	34 (51.52)	57 (68.67)	77 (63.11)	168 (61.99%)	0.095
Corridor	32 (48.48)	26 (31.33)	45 (36.89)	103 (38.01%)	

*IQR: interquartile range; n: number; SP: São Paulo*

### Impact of COVID-19 on Burnout Syndrome and Empathy

The results regarding the impact of COVID-19 on Burnout Syndrome and empathic care of nursing professionals and the data related to the dimensions of the MBI-HSS by professional category are presented in Table 3.

**Table 3:** Impact of the COVID-19 pandemic on Burnout Syndrome and empathy, and levels of the Maslach Burnout Inventory - Human Services Survey dimensions, by professional category, in the Emergency Unit, São Paulo, São Paulo, Brazil, 2021

Item	Answer	Professional Category				*p* value
		Nurse	Nursing technician	Nursing aide	Total	
Impact of the pandemic on BS, n (%)	No	3 (13.04)	4 (14.29)	5 (12.20)	12 (13.04)	0.969
	Yes	20 (86.96)	24 (85.71)	36 (87.80)	80 (86.96)	
	Decreased	1 (5.00)	1 (4.17)	3 (8.33)	5 (6.25)	0.780
	Increased	19 (95.00)	23 (95.83)	33 (91.67)	75 (93.75)	
Impact of the pandemic on empathy, n (%)	No	5 (21.74)	9 (32.14)	17 (41.46)	31 (33.70)	0.271
	Yes	18 (78.26)	19 (67.86)	24 (58.54)	61 (66.30)	
	Decreased	6 (33.33)	2 (10.53)	2 (8.33)	10 (16.39)	0.068
	Increased	12 (66.67)	17 (89.47)	22 (91.67)	51 (83.61)	
Maslach Burnout Inventory-Human Service Survey	Level of Emotional Exhaustion - n (%)					
	Low (≤18)	16 (69.57)	19 (67.86)	31 (75.61)	66 (71.74)	0.485
	Moderate (19-26)	5 (21.74)	5 (17.86)	3 (7.32)	13 (14.13)	
	High (≥27)	2 (8.70)	4 (14.29)	7 (17.07)	13 (14.13)	
	Level of Depersonalization - n (%)					
	Low (≤5)	12 (52.17)	17 (60.71)	26 (63.41)	55 (59.78)	0.861
	Moderate (6-9)	6 (26.09)	5 (17.86)	9 (21.95)	20 (21.74)	
	High (≥10)	5 (21.74)	6 (21.43)	6 (14.63)	17 (18.48)	
	Level of Professional Accomplishment - n (%)					
	Low (≤33)	5 (21.74)	5 (17.86)	7 (17.07)	17 (18.48)	0.895
	Moderate (34-39)	3 (13.04)	2 (7.14)	3 (7.32)	8 (8.70)	
	High (≥40)	15 (65.22)	21 (75.00)	31 (75.61)	67 (72.83)	

*BS: Burnout Syndrome; n: number; SP: São Paulo*

Most professionals (80; 86.96%) reported an impact on Burnout Syndrome and, of these, 75 (93.75%) reported an increase. As for empathic behavior, 61 professionals (66.30%) reported that the pandemic had an impact on it, and of these, 51 (83.61%) reported an increase in empathic care.

Most professionals reported a “low level” of Emotional Exhaustion: 66 (71.74%) and Depersonalization: 55 (59.78%), but a “high level” of Professional Accomplishment: 67 (72.83%). The professionals who reported a “high level” of Emotional Exhaustion and Depersonalization were 13 (14.13%) and 17 (18.48%), respectively, and a “low level” of Professional Accomplishment: 17 (18.48%). No significant differences of Burnout Syndrome were observed in relation to the three nursing professional categories.

### Nursing professionals’ empathy self-reported and perceived by patients

The results of the evaluation of empathic care are described in Table 4. There was no evidence of significant difference between self-reported empathy among professional categories, as well as that reported by patients.

**Table 4:** Results of CARE Measure-Nurses (Brazilian version) and CARE Measure (Brazilian version), by professional category, in the Emergency Unit, São Paulo, São Paulo, Brazil, 2021

Measures	Professional Category				*p* value
	Nurse	Nursing technician	Nursing aide	Total	
Professionals' self-reported empathic behavior - CARE Measure-Nurses					
Mean (standard deviation)	37.57 (7.80)	41.10 (4.82)	40.36 (6.40)	39.89 (6.44)	0.2056
Patients' self-reported empathic behavior - CARE Measure					
Mean (standard deviation)	35.94 (10.51)	39.31 (9.57)	38.77 (8.62)	38.25 (9.45)	0.1294

*CARE: Consultation and Relational Empathy; SP: São Paulo.*

The patients had the following distribution of medical diagnoses grouped into infection (38; 14%), neurological (46; 17%), COVID-19 (42; 15.5%), cardiovascular (30; 11.1%), general gastroenterology (30; 11.1%), and other diagnoses (98; 36.2%).

Table 5 describes the results for patient perceived empathy and medical diagnosis categories, with the infection category as the reference for comparison, as it had the highest perceived empathy (39.8; 95% CI: 36.6 - 43.3). The only evidence of a significant difference was for neurological diagnoses, with lower perceived empathy (34.9; 95% CI: 32.4 - 37.7; p = 0.025). For patients with a COVID-19 diagnosis, the estimated perceived empathy was 39.0; 95% CI: 36.0 - 42.3.

**Table 5:** Patients’ perception of professional empathy, according to the groups of medical diagnoses, in the Emergency Unit, São Paulo, São Paulo, Brazil, 2021

Diagnosis group	Estimated mean (IC 95%)	*p* value
Other	39.4 (37.3; 41.5)	0.825
General gastroenterology	36.1 (31.8; 41.0)	0.211
Cardiovascular	37.0 (33.6; 40.7)	0.260
COVID-19	39.0 (36.0; 42.3)	0.740
Neurological	34.9 (32.4; 37.7)	0.025
Infection	39.8 (36.6; 43.3)	Reference

*IC 95%: Interval of Confidence of 95%.*

### Correlation of Burnout Syndrome dimensions with empathy self-reported by professionals and perceived by patients

In evaluating the correlation between the dimensions of the Burnout Syndrome and the empathy self-reported by the professionals, a significant, negative, and weak association was observed between the empathic behavior evaluated by the professionals and Emotional Exhaustion and Depersonalization, whose values were -0.32 and -0.26, respectively, that is, the greater the self-reported empathy, the lower the Emotional Exhaustion and Depersonalization, and vice-versa. In relation to self-reported empathy and Professional Accomplishment, this presented a significant, positive and moderate association (0.49), that is, the greater the empathy, the greater the Professional Accomplishment and vice-versa.

On the other hand, the correlation between empathy perceived by patients and Emotional Exhaustion (-0.05) and Depersonalization (0.01) was not significant or almost non-existent. As for the empathy perceived by the patients and Professional Accomplishment, there was a significant, positive and weak association (0.22), that is, the greater the empathy perceived by the patient the greater the Professional Accomplishment and vice-versa.

## DISCUSSION

This study investigated the impact of the COVID-19 pandemic on the Burnout Syndrome and empathy of nursing professionals in a public emergency unit.

Although most professionals reported that there was an impact of the COVID-19 pandemic increasing Burnout Syndrome, the results of this study showed that, overall, the levels of Emotional Exhaustion and Depersonalization were low, and the level of Professional Accomplishment was high (positive results).

The results of this study differ from the literature data published on the current pandemic, with most studies pointing to a negative impact on the mental health of healthcare professionals^(3,10-13)^. A study conducted with nurses and physicians between January and February 2020, in China, showed high levels of distress, depression, anxiety, and insomnia^(31)^. A systematic review conducted by WHO, with studies published between December 2019 and February 2021, evidenced that, regardless of geographic location, health professionals presented, in addition to the symptoms mentioned above, fear, Burnout Syndrome, and post-traumatic stress disorder^(32)^.

Few studies used the MBI-HSS instrument in the current pandemic, making comparisons of the Burnout Syndrome of the nursing team in emergency units difficult. However, results contrary to the present research were found in a systematic review and meta-analysis study that analyzed publications from January 1st to November 15th, 2020, about the Burnout Syndrome among nurses, pointing to a higher prevalence of Emotional Exhaustion (34.1%), followed by Depersonalization (12.6%) and lack of Professional Accomplishment (15.2%). This study also revealed the main risk factors for Burnout Syndrome among nurses: younger age, less social support, low preparedness of family and colleagues to face COVID-19, increased perceived threat of COVID-19, longer working time in quarantine areas, in a high-risk environment, in hospitals with inadequate and insufficient material and human resources, higher workload and lower level of training^(8)^.

Another study showed that women in nursing had a higher risk of negative impact on mental health^(32)^. In the present study, none of the demographic variables of the participants showed a significant difference for occurrence of Burnout Syndrome: most of the participants were women (79; 85.87%), whose is common among nursing professionals in health services in Brazil^(10,23,33-34)^, professional experience (65.22% had graduated over 11 years ago) and age (mean of 42 years). These characteristics may be related to a greater personal and professional maturity.

In a study conducted among emergency service nurses in Belgium comparing Burnout Syndrome in the periods before and after the beginning of the COVID-19 pandemic, there was a significant difference in the high risk of Professional Accomplishment (5.71, p = 0.017), with an increase from 23.3% before to 33.4% post-pandemic, but not in the high risk of Emotional Exhaustion and Depersonalization. For the latter two dimensions, there was a slight decrease after the pandemic (high risk for Emotional Exhaustion from 50.9% to 45.8%; high risk for Depersonalization from 59.1% to 55.5%)^(35)^.

In this study, there was no comparison between the periods before and after the start of the COVID-19 pandemic, however, the results of this study on Burnout Syndrome during the pandemic are better than those cited in the Belgium study.

The critical levels of the Burnout Syndrome dimensions were high level of Depersonalization at 18.48% and low Professional Accomplishment at 18.48%, followed by high Emotional Exhaustion at 14.13%. However, nursing technicians and aides had higher levels of Emotional Exhaustion (14.29% and 17.07%, respectively), when compared to nurses (8.70%), which may be related to these nursing categories, for spending most of the time at the bedside with the patient, developing actions in a direct and continuous relationship, associated with stress factors in the fight against the COVID-19 pandemic, such as higher workload, insomnia, fear, pace of the virus dissemination, lack of support and uncertainty about epidemiological issues^(36)^.

Most participants of the present study reported having only one employment relationship, unlike the study in a public emergency service of Rio de Janeiro, in which the majority (56.76%) reported having more than one employment relationship and high levels of Emotional Exhaustion and Depersonalization^(10)^. Having an employment relationship can be considered a positive characteristic for not developing Burnout Syndrome, corroborating the data analyzed in a study of a public emergency room in Minas Gerais^(37)^.

A study conducted with nurses from 40 public health institutions in São Paulo revealed that institutions with the worst working conditions resulted in high levels of Emotional Exhaustion and Depersonalization and low levels of Professional Accomplishment, unlike those with favorable conditions, that is, autonomy, organizational support and control over the environment are important factors that interfere with Burnout Syndrome^(38)^. Thee aspects were not evaluated in this study.

A cross-sectional study carried out with nurses in an emergency service corroborates the findings of this study, presenting a high score for Depersonalization and a low score for Professional Accomplishment^(10)^. Another study in an emergency unit with nurses and nursing technicians also presented depersonalization as the most critical dimension^(37)^. It is known that Depersonalization is evidenced by the professional’s behavior, that is, distancing and negligent treatment towards the patients, as a way to relieve the weariness^(39)^, and this dimension of the Burnout Syndrome can compromise the professional’s empathy, since it requires guarantee of human presence and attention in the experience with the other.

In the present study, nursing professionals reported that COVID-19 had an impact on increasing empathic care, which is congruent with the findings of another study in the context of COVID-19 that emphasizes that the professional’s understanding of the situation experienced by the patient is a coping strategy against Burnout Syndrome, since paying attention to the feelings of others increases levels of professional well-being and, consequently, Professional Accomplishment^(40)^.

Self-reported empathy showed no difference between the professional categories (p=0.2056), unlike the findings of another study with nursing professionals working in urgency and emergency services that showed that nurses are more empathic than nursing technicians (p=0.039)^(41)^.

Although the correlation between the empathy self-reported by the professional and that perceived by the patient is not the objective of this study, both have their relevance, empathy being a unique element not only for nursing professionals but also for patients^(42)^.

This study showed a negative and weak correlation in the Emotional Exhaustion and Depersonalization dimensions of Burnout Syndrome with self-reported empathy. A study carried out with primary care health professionals evidenced that empathy was a protective factor against Burnout Syndrome^(20)^. Another study with primary care physicians also showed that high levels of self-reported empathy were associated with low levels of Burnout Syndrome^(43)^. A study with emergency physicians also showed a weak negative correlation between self-reported empathy and Burnout Syndrome^(44)^.

A cross-sectional study carried out with nurses and users assisted by these professionals, in primary care in Porto Alegre, evaluated the empathy self-reported by the professionals and perceived by the users and the impact of occupational stress, and the users evaluated that professionals with higher levels of stress presented less empathy^(45)^. In this study, there was evidence that the greater the empathy perceived by the patient, the greater the Professional Accomplishment and vice-versa. Therefore, it is understood that self-reported empathy is related to the way the individual is in his occupational environment, and may be a protective factor for Burnout Syndrome, since the professional, by understanding the importance of empathy in care, deals better with the patient and with problematic situations at work^(20,40)^.

It is important to emphasize that empathy is not only related to the ability to understand patients’ experiences and feelings, but also to the ability to communicate their feelings through behavior, whose essence is altruistic, resulting in greater adherence to treatment and patient satisfaction^(46)^. Moreover, professionals develop a closer relationship with their patients and care that is full of energy, enthusiasm^(43)^, and engagement^(47)^, with the ability to transform the extremely emotional and stressful experience, through resilient emotional management and consequent progressive adaptation^(48)^.

No studies were found evaluating Burnout Syndrome and empathy in the nursing team in the Emergency Unit, in the context of COVID-19, making comparisons difficult. The current study was carried out between October 2020 and March 2021, when, in Brazil, there was a substantial drop in the number of cases and deaths of patients with COVID-19^(49)^, which may have influenced the results.

### Study limitations

The study was carried out in a single public emergency unit, with members of the nursing team, and it is not possible to generalize the results. The cross-sectional study design has limitations in relation to changes over time; furthermore, risk factors for Burnout Syndrome and empathic care were not evaluated.

### Contributions to the field

Professional empathy is associated with low levels of Emotional Exhaustion and Depersonalization and high Professional Accomplishment, being a fundamental factor in the mental health of professionals and in the quality of care provided to patients.

Thus, the empathic and communicational skills of professionals may be essential for the nursing work to be meaningful and rewarding and, therefore, should be developed and improved for greater involvement and well-being of professionals and possible positive repercussions for patients and the organization as a whole.

## CONCLUSIONS

The COVID-19 pandemic impacted Burnout Syndrome, however most professionals reported “low level” Burnout Syndrome for Emotional Exhaustion and Depersonalization and high level of Professional Accomplishment. The professionals reported a positive impact on empathic care.

There was evidence of a significant association: negative - the higher the self-reported empathy, the lower the level of Emotional Exhaustion and Depersonalization; and positive - the higher the self-reported empathy, the higher the Professional Accomplishment. Regarding the empathy perceived by the patients, there was no significant association between the Emotional Exhaustion and Depersonalization dimensions, but there was evidence of a significant positive association with Professional Accomplishment.
